# “What Is Hidden behind the Mask?” Facial Emotion Recognition at the Time of COVID-19 Pandemic in Cognitively Normal Multiple Sclerosis Patients

**DOI:** 10.3390/diagnostics12010047

**Published:** 2021-12-27

**Authors:** Stefano Ziccardi, Francesco Crescenzo, Massimiliano Calabrese

**Affiliations:** 1 Neurology Section, Department of Neuroscience, Biomedicine and Movement Sciences, University of Verona, 37134 Verona, Italy; 2 Neurology Unit, Mater Salutis Hospital, AULSS 9 Scaligera, 37045 Verona, Italy; francescocrescenzo89@gmail.com

**Keywords:** multiple sclerosis, facial emotion recognition, COVID-19, face masks, social cognition

## Abstract

Social cognition deficits have been described in people with multiple sclerosis (PwMS), even in absence of a global cognitive impairment, affecting predominantly the ability to adequately process emotions from human faces. The COVID-19 pandemic has forced people to wear face masks that might interfere with facial emotion recognition. Therefore, in the present study, we aimed at investigating the ability of emotion recognition in PwMS from faces wearing masks. We enrolled a total of 42 cognitively normal relapsing–remitting PwMS and a matched group of 20 healthy controls (HCs). Participants underwent a facial emotion recognition task in which they had to recognize from faces wearing or not surgical masks which of the six basic emotions (happiness, anger, fear, sadness, surprise, disgust) was presented. Results showed that face masks negatively affected emotion recognition in all participants (*p* < 0.001); in particular, PwMS showed a global worse accuracy than HCs (*p* = 0.005), mainly driven by the “no masked” (*p* = 0.021) than the “masked” (*p* = 0.064) condition. Considering individual emotions, PwMS showed a selective impairment in the recognition of fear, compared with HCs, in both the conditions investigated (“masked”: *p* = 0.023; “no masked”: *p* = 0.016). Face masks affected negatively also response times (*p* < 0.001); in particular, PwMS were globally hastier than HCs (*p* = 0.024), especially in the “masked” condition (*p* = 0.013). Furthermore, a detailed characterization of the performance of PwMS and HCs in terms of accuracy and response speed was proposed. Results from the present study showed the effect of face masks on the ability to process facial emotions in PwMS, compared with HCs. Healthcare professionals working with PwMS at the time of the COVID-19 outbreak should take into consideration this effect in their clinical practice. Implications in the everyday life of PwMS are also discussed.

## 1. Introduction

Multiple sclerosis (MS) is an immune-mediated disease of the central nervous system characterized by a chronic and progressive course related to white matter and gray matter demyelination, neurodegeneration, and axonal injury processes [1,2,3].

One of the most common clinical symptoms in people with MS (PwMS) is cognitive impairment, affecting from 34% to 65% of patients suffering from memory, attention, information processing speed, executive functioning, and social cognition deficits [4,5,6]. During the last decade, in the MS literature, a great interest has emerged around the concept of the social cognition domain [7,8], which highly depends on facial emotion recognition, the ability to identify human emotions from facial expression [9]. Deficits in facial emotion recognition have been described in MS populations in many recent studies, even independently from global cognitive impairment [10,11,12]. We demonstrated the lower performance of cognitively normal MS individuals in a facial emotion recognition task, compared with controls [13], and the persistence of this alteration after 3 years of follow-up [14]. Moreover, previous MS studies highlighted a specific deficit in the recognition of negative emotions; in particular, studies agreed that PwMS are mainly impaired in the recognition of fear and anger [7,13,15,16].

The ongoing pandemic of coronavirus disease 2019 (COVID-19) has changed people’s entire life, in particular their social interactions: prolonged social contacts have been forbidden, and, when allowed to physically meet, people must wear surgical face masks since the first months of 2020, following the recommendations of health authorities. The obligation of wearing masks had surely positive medical benefits in the virus spread [17,18]; however, the ability to recognize which emotion people are experiencing by looking at their faces was hindered, since individuals were forced to meet other people without the possibility to see half of the human face, in particular the nose, the lips, the cheeks. These are considered crucial areas for facial emotion recognition, mainly disgust and happiness [19]; therefore, the use of masks may lead to inaccurate face processing which can crucially affect social everyday interactions [20,21,22,23]. Detailed processing of human faces is already more difficult with partial occlusions commonly used in everyday life (i.e., sunglasses, scarfs, headdresses, veils) [24,25,26]; consequently, it might be even more altered when people wear masks that cover between 60% and 70% of the entire face, depending on shapes and sizes [27], forcing people to ground the emotion identification mainly on cues from the eyes [28]. Some studies have been conducted in healthy controls on this topic since the beginning of the COVID-19 pandemic, highlighting the lower accuracy in the emotion recognition of faces wearing masks [27,29,30,31]. However, a non-impaired performance was described in adults for anger and happiness [32], and in children for anger, sadness, and fear [33]. Mixed results are provided, and up to now, it is not completely clear the specific impact of masks in processing different face expressions.

Considering that PwMS already showed an impaired facial emotion recognition in normal conditions while seeing the whole face [13,14], the present preliminary study aimed at investigating the ability of cognitively normal PwMS, compared with matched healthy controls, in recognizing emotions from facial expressions when stimuli are covered with face masks, as typical during the time of COVID-19 pandemic. To the best of our knowledge, no similar study has been conducted so far including individuals with MS; therefore, we aimed at exploratively evaluating the impact of face masks in the process of facial emotion recognition both at a global level and considering specific emotions, measuring whether the effect of mask wearing is similar between PwMS and healthy controls. Moreover, we investigated in both PwMS and controls some confusion patterns in the attribution of specific emotions with other emotional states.

We hypothesized that, in the normal condition of seeing fully visible human faces, PwMS would show lower performance than HCs in the recognition of negative emotions, mainly fear and anger, due to the MS alterations that are clearly described in the abovementioned studies; however, we expected that the new condition of recognizing emotions and expressions from half-covered faces typical of the COVID-19 period could be of the same considerable and comparable difficulty for both PwMS and HCs.

## 2. Materials and Methods

### 2.1. Study Design and Participants

During the time of the COVID-19 pandemic, for the present study, we enrolled 42 consecutive cognitively normal PwMS (27 females, mean ± SD age = 33.5 ± 8.4 years, mean ± SD education = 16.0 ± 3.0 years, mean ± SD disease duration = 5.8 ± 4.4 years). PwMS had a short disease duration (median [IQR] = 4.5 [4.0] years) and a low physical disability (median [IQR] EDSS = 1.25 [2.5]). At the time of testing, all PwMS but one were under immunomodulatory therapy: 10 were treated with dimethyl fumarate, 9 with cladribine, 8 with natalizumab, 6 with ocrelizumab, 5 with fingolimod, 2 with teriflunomide, and 1 with azathioprine. Inclusion criteria for PwMS were diagnosis of relapsing–remitting MS [34], no relapse or steroid treatment in the month before the cognitive evaluation, no other comorbidities, no substance abuse, normal or corrected to normal vision, and absence of cognitive impairment. The cognitive performance of PwMS was evaluated by means of the Brief Repeatable Battery of Neuropsychological Tests (BRB-NT) [35] and the Stroop Test (ST) [36]. According to the classification described in our previous work [37], to be classified as cognitively normal, each subject had to achieve corrected scores above the clinical cut-off (5th percentile) in all the cognitive subtests administered (BRB-NT and ST). The emotional state was also evaluated through the Depression, Anxiety, and Stress Scale 21 (DASS-21) [38].

We also recruited a group of 20 healthy controls (HCs) (13 females, mean ± SD age = 36.8 ± 14.4 years, mean ± SD education = 17.4 ± 2.8 years), matched with PwMS for age (*p* = 0.09), education (*p* = 0.36), and gender (*p* = 0.82), that were tested with the same facial emotion recognition task. Similarly, inclusion criteria for HCs were no concomitant pathologies that could affect cognition, no substance abuse or other prior or concomitant medications, and normal or corrected-to-normal vision.

All PwMS were recruited and tested at the MS Center of the Verona University Hospital (Verona, Italy). The study was conducted in accordance with the Declaration of Helsinki (2013), approved by the local Ethics Committee (Protocol Code 66,418; date of approval 25 November 2019) and informed consent was collected.

### 2.2. Experimental Task—Stimuli and Procedure

The facial emotion recognition (FER) task was administered to all the participants. The task was experimentally prepared by selecting 36 black-and-white photos of different actors displaying one of the six basic emotions (happiness, anger, fear, sadness, surprise, disgust). The stimuli were selected from the Karolinska Directed Emotional Faces database (KDEF) [39], which includes well-validated stimuli for facial emotion recognition. Each emotion was presented on a screen six times (6 emotions for 6 times each = 36 stimuli), as described in previous works from our group [13,14]. Participants had to see each image twice, one time with the surgical mask (graphically applied covering the lower part of the face including nose and mouth), and one time without the surgical mask, for a total of 72 images (6 actors × 6 emotions × 2 conditions (“mask” vs. “no mask”) = 72 stimuli). Images were presented in a pseudorandomized order, in which the conditions of “mask”/“no mask” and the emotion presented were always balanced (never more than 3 “masked”/“no masked” images in a row and never more than 2 images with the same emotion in a row). Participants were asked to choose which emotion each face was expressing, and responses were registered. Response times (RTs) were also recorded for each picture; however, participants were instructed to take all the time they needed. As a control task, participants were also asked to indicate whether each image was showing a female or a male face, in order to verify adequate compliance and avoid random answers. One point was assigned for each correct response, while no points were assigned for incorrect responses. The entire procedure lasted around 10–15 min.

### 2.3. Statistical Analyses

Statistical analyses were performed using SPSS statistic software (SPSS Inc., Chicago, IL, USA, version 24). The chi-squared test was applied to compare the female/male ratio between PwMS and HCs. Shapiro–Wilk tests were performed to evaluate the normality distribution of the variables, in order to apply parametric or non-parametric tests when appropriate. Correlation analyses were performed to evaluate the association between emotional state and facial emotion recognition performance (Pearson and Spearman correlations methods were used appropriately). *t*-tests for independent samples and Mann–Whitney tests were performed, when appropriate, to evaluate the difference in the facial emotion recognition accuracy and RTs between the two groups of PwMS and HCs, while *t*-tests for paired samples and Wilcoxon tests were performed, when appropriate, to evaluate the difference in the facial emotion recognition accuracy and RTs within the two groups comparing “masked” and “no masked” conditions. Group accuracy is expressed as the average of correct responses of individuals, while group RTs as the average of response times of individuals. Means ± SDs and median [IQR] were provided for continuous variables, considering mean ± SD in parametric analyses and median [IQR] in non-parametric analyses. Effect sizes are reported in terms of Cohen’s *d* for parametric analyses, and in terms of *r* for non-parametric analyses. A *p*-value less than 0.05 was considered significant.

## 3. Results

### 3.1. Facial Emotion Recognition Task—Correct Answers (Accuracy)

No associations were found between emotional state (DASS-21) and FER accuracy scores (global score, “masked” condition total score, “no masked” condition total score, individual emotions scores) (all *p* > 0.05).

**Figure 1:**
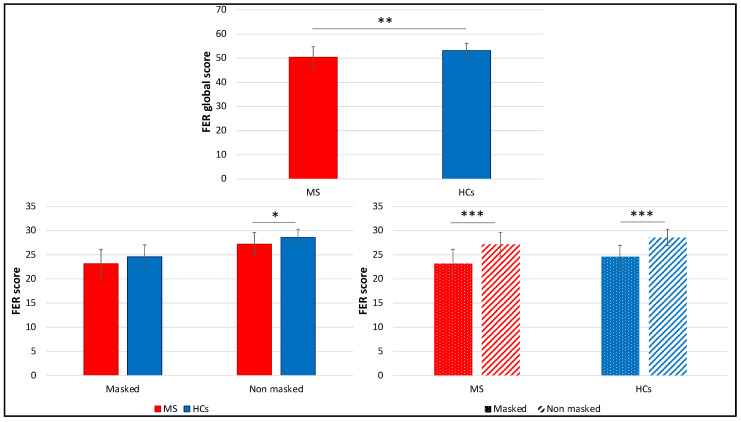
Comparison between MS and HCs FER global accuracy and scores in the two conditions (“masked” vs. “no masked”). Means and SDs are reported. * *p* < 0.05; ** *p* < 0.01; *** *p* < 0.001.

Considering together the “masked” and the “no masked” condition of the FER task, PwMS reached on average a significantly lower global score (50.4 ± 4.4), compared with the control group (53.2 ± 3.0) (*t*(60) = −2.93, *p* = 0.005, *d* = 0.70). Evaluating the two conditions separately, results showed a statistically significant difference for the “no masked” condition (MS: 27.2 ± 2.4, HCs: 28.6 ± 1.7, *t*(60) = −2.37, *p* = 0.021, *d* = 0.64), while in the “masked” condition, we observed a trend of significance (MS: 23.2 ± 2.9, HCs: 24.6 ± 2.5, *t*(60) = −1.83, *p* = 0.064, *d* = 0.50). In general, both PwMS and HCs showed a significantly lower accuracy in the “masked”, compared with the “no masked” condition (MS: *t*(41) = −8.64, *p* < 0.001, *d* = 1.33; HCs: *t*(19) = −5.98, *p* < 0.001, *d* = 1.34) (Figure 1).

**Figure 2:**
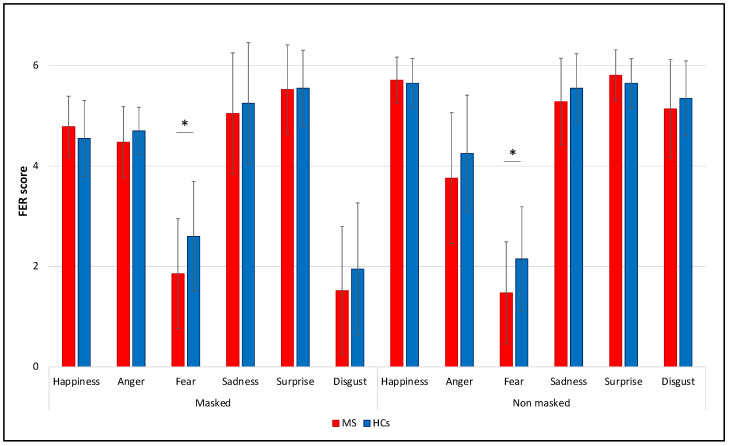
Comparison between MS and HCs FER scores considering each individual emotion in the two conditions (“masked” vs. “no masked”). Means and SDs are reported. * *p* < 0.05.

Regarding the accuracy within each individual emotion in the two conditions, the unique significant difference between PwMS and HCs was found for fearful faces, in which PwMS identified correctly fewer images than HCs in both the “masked” (MS: 2.0 [2.0], HCs: 3.0 [1.25]), *U* = 273.5, *p* = 0.023, *r* = 0.30) and the “no masked” (MS: 1.0 [1.0], HCs: 2.0 [2.0]), *U* = 267, *p* = 0.016, *r* = 0.31) conditions. All the other comparisons for individual emotions in terms of accuracy were not significant (all *p* > 0.05) (Figure 2).

**Figure 3:**
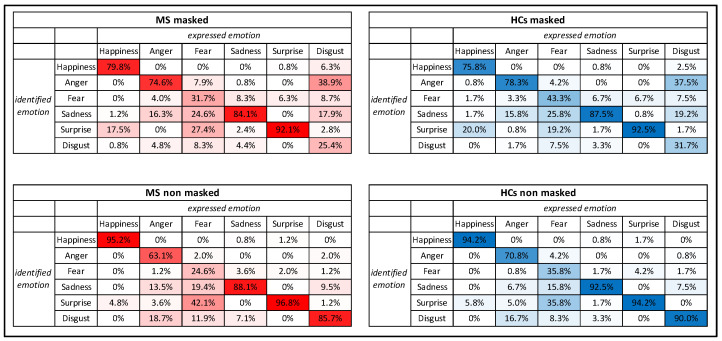
Facial emotion recognition confusion matrices indicating the percentage of correct and wrong answers for MS and HCs in the two conditions. Rows indicate the answer pronounced by the participants, while columns indicate the emotion that was effectively presented. **Colors in-tensity reflects the accuracy: the more intense the colors, the higher the percentage of correct answers**.

In order to qualitatively investigate these misinterpretations, confusion matrices indicating the percentage of correct answers or mistakes were built for each condition in the two groups. Results from the confusion matrices showed the more common correct responses and errors in the facial emotion recognition task for both the two groups; evaluating correct answers, accuracy is similar between PwMS and HCs except for fearful faces in which PwMS showed a lower percentage of correct answers (as stated above and showed in Figure 2). However, results showed that, in the “masked” condition, disgust and fear were the most difficult emotion to identify for both PwMS and HCs. The most typical mistakes were to confound disgust with anger (more frequently, MS: 38.9%, HCs: 37.5%) or with sadness (more rarely, MS: 17.9%, HCs: 19.2%), while fear was confused mostly with surprise (MS: 27.4%, HCs: 19.2%) and sadness (MS: 24.6%, HCs: 25.8%), and happiness were often misidentified as surprise (MS: 17.5%, HCs: 20.0%). In the “no masked” condition, as observed before in Figure 2, disgust was the emotion that gained the highest difference of correct response, compared with the “masked” condition (MS: 85.7% vs. 25.4%, HCs: 90.0% vs. 31.7%), followed by happiness (MS: 95.2% vs. 79.8%, HCs 94.2% vs. 75.8%); fear was the emotion with the lowest accuracy for all the participants (MS: 24.6%, HCs 35.8%), typically misjudged with surprise (more frequently MS: 42.1%, HCs 35.8%) and sadness (more rarely, MS: 19.4%, HCs 15.8%), followed by anger (MS: 63.1%, HCs 70.8%) often mismatched with disgust (MS: 18.7%, HCs 16.7%). When seeing full faces, both PwMS and HCs showed the highest correct percentage of responses in the identification of surprise and happiness (accuracy higher than 94%). The more common mistakes in the emotion recognition process are presented in detail in Figure 3.

Comparing the accuracy between the two conditions for each individual emotion separating PwMS and HCs, results showed that PwMS were significantly less accurate in recognizing emotions from “masked” faces, compared with “no masked” faces, showing happiness (“masked”: 5.0 [0.75], “no masked”: 6.0 [1.0], *Z* = −5.10, *p* < 0.001, *r* = 0.56), surprise (“masked”: 6.0 [1.0], “no masked”: 6.0 [0], *Z* = −2.08, *p* = 0.032, *r* = 0.23) and disgust (“masked”: 1.0 [2.5], “no masked”: 5.0 [1.0], *Z* = −5.68, *p <* 0.001, *r* = 0.62), while were significantly more accurate in recognizing emotions from “masked” faces, compared with “no masked” faces, showing anger (“masked”: 5.0 [1.0], “no masked”: 4.0 [1.0], *Z* = −3.50, *p* < 0.001, *r* = 0.38). All the other comparisons were not significant (all *p* > 0.05).

**Figure 4:**
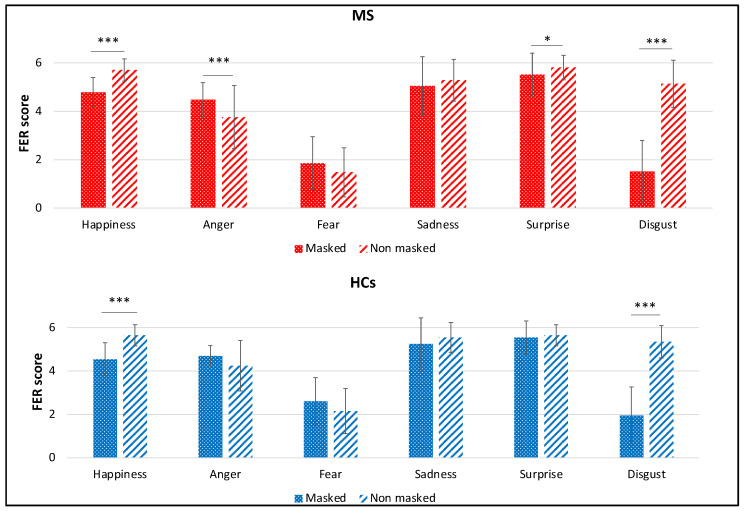
Comparison between the accuracy considering individual emotions in the two conditions (“masked” vs. “no masked”) in MS and HCs. Means and SDs are reported. * *p* < 0.05; *** *p* < 0.001.

In the HCs group, results showed a significant negative effect on the accuracy of the surgical mask in faces showing happiness (“masked”: 5.0 [1.0], “no masked”: 6.0 [1.0], *Z* = −3.51, *p* < 0.001, *r* = 0.55) and disgust (“masked”: 2.0 [2.0], “no masked”: 5.5 [1.0], *Z* = −3.95, *p* < 0.001, *r* = 0.63). All the other comparisons were not significant (all *p* > 0.05). Detailed results are reported in Figure 4.

### 3.2. Facial Emotion Recognition Task—Response Times (Speed)

No associations were found between emotional state (DASS-21) and FER RTs (global time, “masked” condition total time, “no masked” condition total time, individual emotions total times) (all *p* > 0.05).

**Figure 5:**
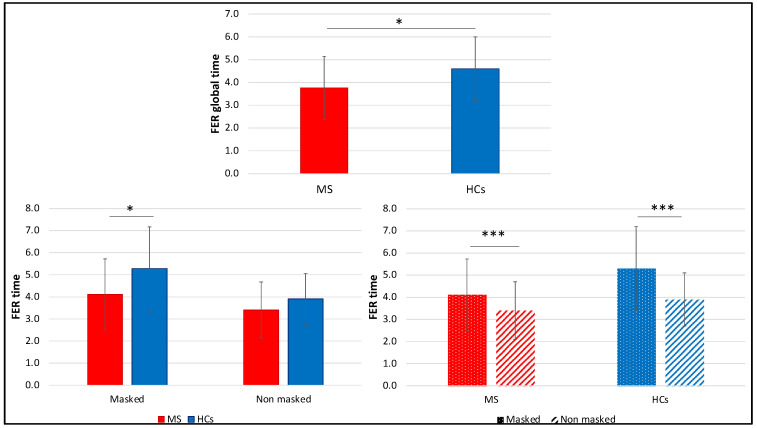
Comparison between MS and HCs FER global averaged RT and RTs in the two conditions (“masked” vs. “no masked”). RTs are expressed in seconds, means and SDs are reported. * *p* < 0.05; *** *p* < 0.001.

Considering together the “masked” and the “no masked” condition of the FER task, PwMS were on average significantly faster in providing their answer (3.6 [1.7] s), compared with the control group (4.4 [1.9] s), *U* = 270, *p* = 0.024, *r* = 0.30. However, evaluating the two conditions separately, results showed a statistically significant difference for the RTs in the “masked” condition (MS: 4.0 [1.7] s, HCs: 5.3 [2.2] s, *U* = 255.5, *p* = 0.013, *r* = 0.32), while in the “no masked” condition we observed a trend of significance (MS: 3.2 [1.9] s, HCs: 3.5 [1.9] s, *U* = 308.5, *p* = 0.093, *r* = 0.21). In general, both PwMS and HCs showed significantly higher RTs in the “masked”, compared with the “no masked” condition (MS: *Z* = −4.63, *p* < 0.001, *r* = 0.51; HCs: *t*(19) = 4.36, *p* < 0.001, *d* = 0.98) (Figure 5).

**Figure 6:**
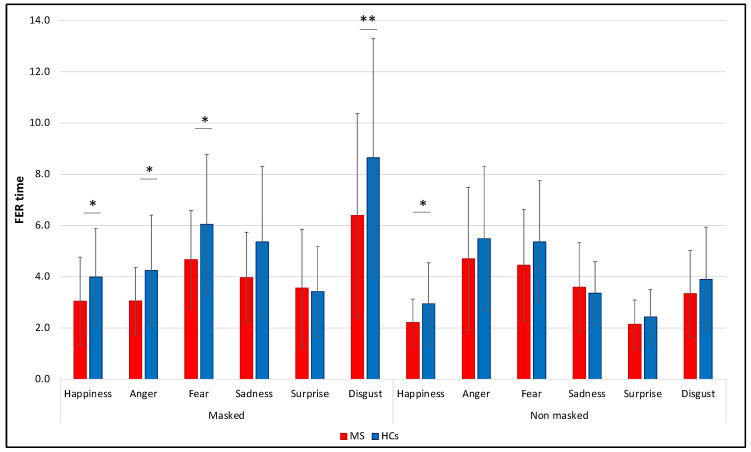
Comparison between MS and HCs of FER averaged RTs considering each individual emotion in the two conditions (“masked” vs. “no masked”). RTs are expressed in seconds, means and SDs are reported. * *p* < 0.05; ** *p* < 0.01.

Regarding the RTs within each individual emotion in the two conditions, significant differences were found in the “no masked” condition for happy faces, in which PwMS were faster (1.8 [1.0] s) than HCs (2.6 [2.2] s) (*U* = 288.5, *p* = 0.048, *r* = 0.25), while in the “masked” condition the same pattern of significance was observed for happy (MS: 2.5 [1.6] s, HCs: 3.6 [1.8] s, *U* = 260, *p* = 0.016, *r* = 0.31); angry (MS: 2.8 [2.1] s, HCs: 3.6 [2.4] s, *U* = 278, *p* = 0.032, *r* = 0.27), fearful (MS: 4.3 [2.5] s, HCs: 5.9 [2.9] s, *U* = 282.5, *p* = 0.038, *r* = 0.26), and disgusted faces (MS: 5.3 [3.3] s, HCs: 8.0 [2.1] s, *U* = 239, *p* = 0.006, *r* = 0.35). All the other comparisons for individual emotions in terms of RTs were not significant (all *p* > 0.05) (Figure 6).

Comparing the RTs between the two conditions for each individual emotion, results showed that PwMS took longer times in recognizing emotions from “masked” faces, compared to “no masked” faces, showing happiness (“masked”: 2.5 [1.6], “no masked”: 1.8 [1.0], *Z* = −3.65, *p* < 0.001, *r* = 0.41), surprise (“masked”: 2.8 [2.5], “no masked”: 1.8 [1.0], *Z* = −5.11, *p* < 0.001, *r* = 0.57), and disgust (“masked”: 5.3 [3.3], “no masked”: 3.0 [1.8], *Z* = −5.19, *p* < 0.001, *r* = 0.58), while took less time in recognizing emotions from masked faces, compared with “no masked” faces, showing anger (“masked”: 2.8 [2.1], “no masked”: 4.1 [2.9], *Z* = −4.56, *p* < 0.001, *r* = 0.51). All the other comparisons were not significant (all *p* > 0.05).

**Figure 7:**
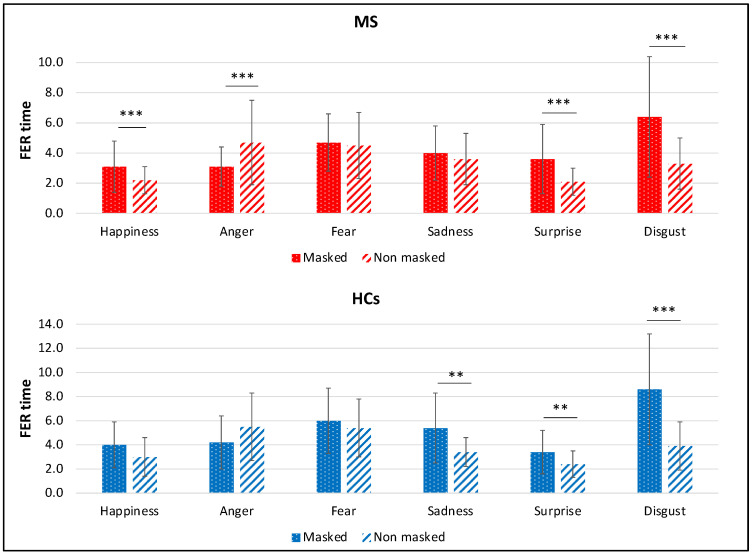
Comparison between the averaged RTs considering individual emotions in the two conditions (“masked” vs. “no masked”) in MS and HCs. RTs are expressed in seconds, means and SDs are reported. ** *p* < 0.01; *** *p* < 0.001.

In the HCs group, results showed a negative effect on RTs of the surgical mask in faces showing sadness (“masked”: 4.6 [3.9], “no masked”: 3.2 [1.8], *Z* = −2.80, *p* = 0.005, *r* = 0.44), surprise (“masked”: 2.9 [2.1], “no masked”: 2.2 [0.8], *Z* = −3.21, *p* = 0.001, *r* = 0.51), and disgust (“masked”: 8.0 [2.1], “no masked”: 3.3 [2.2], *Z* = −3.92, *p* < 0.001, *r* = 0.62). All the other comparisons were not significant (all *p* > 0.05). Detailed results are reported in Figure 7.

## 4. Discussion

The aim of the present study was to investigate, for the first time, the facial emotion recognition ability in a group of cognitively normal PwMS, compared with a matched control group, when forced to look at human faces wearing masks, as utilized daily since 2020 due to the COVID-19 pandemic. On the overall facial emotion recognition performance, despite the classification of “cognitive normality”, PwMS showed a significantly lower performance compared with controls, suggesting a specific impairment in this social cognition domain independent from other cognitive alterations, as reported in previous studies [10,11,12,13]. As expected, for both PwMS and HCs the emotion identification process suffered from the use of the face mask, which strongly negatively influenced the accuracy, as demonstrated by the very large effect sizes reported. However, when images were differenced for the “masked” vs. “no masked” condition, the MS group showed a significantly lower score, compared with the control group in the “no masked” condition, while the difference was lower between PwMS and HCs in the “masked” images, reporting a trend of significance. Therefore, our data seem to suggest that face masks negatively affect the emotion identification in both individuals with MS and controls, but the difference between MS and HCs groups was more driven by the “no masked” condition, in which PwMS achieved significantly lower scores, while the non-usual condition of identifying facial expression in individuals that are wearing face masks seems to share approximately the same difficulty in both PwMS and HCs, as we hypothesized.

Moreover, looking at the response times results, as reported for accuracy, the use of masks on human faces reflects a substantial significant increment of response times for both PwMS and HCs, making the identification process considerably more complicated and more time consuming. Globally, PwMS were significantly faster than HCs in the pronunciation of the answer, reflecting a response rush; in particular, this difference was more driven by the “masked” condition, in which PwMS took a significantly shorter time to pronounce the response, while HCs needed on average a longer time to interpret the non-usual masked face. By contrast, in the “no masked” condition, the response times of PwMS and HCs were comparable.

Considering separately the six basic emotions, results for both “masked” and “no masked” conditions showed that the selective impairment for PwMS, compared with HCs, was in the accuracy of recognizing fearful faces. This is in line with our previous work in which fear was the most difficult emotion for PwMS to identify, probably due to cortical damage in the amygdala, acknowledged as the neural hub for fear identification [13]. These results are also supported by evidence that individuals with amygdala lesions tend to shift the attention in the emotion recognition process away from the eyes [40,41]: given the fact that MS cortical damage could severely affect the amygdala, PwMS could have more difficult in the gaze orientation towards the eyes of fearful faces, leading to impaired identifications, especially when faces to elaborate are wearing masks without providing any other facial cues. In terms of response times, PwMS took a significantly shorter time than HCs, to provide responses for “masked” fearful faces, which could probably reflect the lower accuracy in the recognition of this emotion; moreover, results of significantly faster responses of PwMS were observed also in “masked” angry and disgusted faces and “no masked” happy faces.

Results from the confusion matrices showed the more common correct responses and errors in the emotion recognition task for both groups, which showed a similar pattern of answers. Globally, the identification of fearful faces was the most difficult for both groups and in both conditions, as stated above; in fact, it has been shown that the expression of fear is associated in an equal manner both to the eyes and to the mouth [42]. In the “masked” condition, disgust (confused with anger or, more rarely, sadness) and fear (confused with surprise or sadness) were the most difficult emotions to be identified for both PwMS and HCs, followed by happiness (commonly confused with surprise). In the “no masked” condition, the lowest accuracies in recognition were found for fear (confused with surprise and sadness) and anger (confused with disgust). Comparing the two conditions, primarily disgust and secondly happiness are the emotions that report the higher “surgical mask effect” in terms of the difference between “masked” and “no masked faces”, suggesting how the manifestation of disgust and happiness are highly dependent on the lower part of the face (mouth, cheek, lips, and nose) for correct identification; therefore, the application of face masks affects severely their elaboration, in particular, with disgust, an emotion that without seeing the lower part of the face tends to often be mismatched with anger (according to [27]). Disgusted faces in the “masked”, compared with the “no masked” condition, were also processed for a longer time, compared with all the other emotions, corroborating the great reliance of the expression of this emotion on the lower part of the face; in fact, an interesting study reported that fear and sadness are decoded in the eyes of individuals, while happiness and disgust are interpreted in their mouth [19]. The same conclusion is reported by Spitzer [43], who, in line with our results, emphasized how happiness and disgust are the most difficult emotions to be identified in a “masked” face. Moreover, results also show how fear and anger are confused, respectively, with surprise/sadness and with disgust, highlighting how difficult is for PwMS to correctly recognize and categorize negative emotions, independently from the use of face masks and compared with positive emotions [13,44]. In particular, angry faces seemed actually to reflect a “benefic” effect from the mask, since both PwMS and HCs showed on average a higher accuracy and shorter response times in the “masked” condition with angry faces, probably because this emotion is highly expressed from the higher part of the face (mainly the eyes, the eyebrows, and the corrugator muscle). These results are supported by evidence that showed how different parts of the face are more important for specific emotions; for instance, the mouth is particularly relevant for the identification of happiness, and the eyes are particularly relevant for the identification of anger [42].

Furthermore, we considered independently the two groups, and we compared accuracy and response times for PwMS and HCs. In terms of accuracy, PwMS and HCs showed considerably higher scores in the “no masked” condition when identifying disgusted and happy faces, while PwMS (but not HCs) showed lower scores seeing “masked” vs. “no masked” surprised faces and higher scores seeing “masked” vs. “no masked” angry faces. In the MS group, the same pattern of accuracy was observed for response times, suggesting that some emotions required less time to be identified—namely, PwMS were significantly slower when seeing “masked” vs. “no masked” disgusted, surprised, and happy faces, while the pattern was opposite with angry faces in which the “masked” condition was faster identified. The control group showed the same lower responses as PwMS for the “masked” (compared with “no masked”) disgusted and surprised faces and showed a significantly shorter response speed in front of sad “masked”, compared with “no masked”, faces. A recent study with healthy individuals [27] showed the same difficulty due to the mask, primarily with disgusted faces and secondly with happy faces, as found in the present study, in addition to other significant effects for “masked” sad and angry faces, compared with “no masked” faces. In the study of Carbon [27], no effect of the mask was found for fearful faces, while the confusion matrices of our study showed that fear is the most mismatched emotion; this is probably because the previous study did not include surprised faces, expressions that, in our study, were most confused with fearful expressions. Another recent study [32] corroborates our results for HCs, highlighting that the use of masks that show barely the higher part of the face facilitates the recognition of negative emotion such as anger and fear but negatively affects the recognition of positive emotion such as happiness. In general, as expected, the application of face masks tends to decrease the familiarity of the person seen, with the natural consequence of a more complicated emotional recognition. Additionally, Bani et al. [29] showed a negative mask effect for happy faces, and they also reported the same trend for sad and angry faces.

It is important to highlight that the PwMS enrolled in the present study are all classified as “cognitively normal”; therefore, the lower performance on the FER task could not be ascribed to other cognitive alterations due to MS. Moreover, since the MS sample showed on average a relatively short disease duration from MS diagnosis (~5 years), we demonstrated that the specific difficulty regarding facial emotion recognition also affects patients in the early MS course. Furthermore, it is noteworthy that the performance of facial emotion recognition is not associated with the emotional states of participants in terms of depression, anxiety, and stress, representing consequently a specific cognitive alteration due to the MS activity.

Results from the present study also provide important psychological aspects about using face masks in the COVID-19 pandemic. Since the beginning of 2020, a great number of studies have underlined all the negative psychological effects that the COVID-19 pandemic caused to all individuals worldwide [45,46,47,48,49]. It is of fundamental importance to consider that masks increase the social distance between individuals [32] and influence psychological status, particularly in men that typically feel more negative emotions while wearing face masks, compared with women, and therefore less prone to wear them [50]. This is true for all individuals that, since the beginning of 2020, are facing this situation in which is difficult to understand which emotion other people are experiencing in everyday life. For patients, considering the healthcare setting, the ability to correctly identify and interpret emotions is of fundamental importance in the patient–healthcare professional relation [29,51]. For instance, primary care doctors wearing medical face masks were perceived by patients on average as less empathic, especially when patients were under the same doctor care for a long period of time [52]. Therefore, healthcare professionals need to take into consideration the higher distance to the patients and the alterations in emotional recognition of their patients caused by using face masks in all clinical moments (i.e., communication of a diagnosis, the explanation of the beneficial and side effects of therapies).

Our study is not free from limitations. Firstly, we are aware that the samples of PwMS and HCs are limited and were recruited in the same geographical area; further studies should include a higher number of participants from different MS centers, in order to improve the data generalization. Secondly, to investigate facial emotion recognition abilities, we used a database of static and non-genuine pictures of human faces, and one could argue that this procedure could be far from “real life”; therefore, future studies should also evaluate the performance of emotional recognition using more dynamic assessment, for example, videos and other genuine emotional manifestations including additional information that could provide more details regarding the emotion expressions, such as the tone of the voice, body posture, and gestures.

## 5. Conclusions

In conclusion, preliminary results from the present study showed that individuals with MS and controls were less accurate and took more time to answer when forced to recognize emotions from “masked” faces, compared to the identification of faces without masks. Cognitively normal PwMS achieved a lower performance in facial emotion recognition, compared with a control group, and were also hastier to provide answers. All the participants (both PwMS and HCs) significantly better recognized happy and disgusted faces, and were also faster in identifying disgusted and surprised faces without the masks; however, compared with healthy individuals, PwMS significantly better recognized “no masked” surprised and angry “masked” faces, and were also faster in identifying “no masked” happy and “masked” angry faces. Our group of PwMS showed a selective impairment in the recognition of fear (in both “masked” and “no masked” conditions), and also lower performance with disgust and happiness which are more difficult to be interpreted when the lower part of the face is covered. Healthcare professionals should consider these results, and the consequent implication of the more complicated emotive connection due to the use of face masks at the time of COVID-19.

## Data Availability

The data supporting the findings of this study are available from the corresponding author on reasonable request.
